# Auditory Stimulation of Slow‐Wave Sleep Promotes Recovery after Brain Injury in an Animal Model

**DOI:** 10.1002/ana.78234

**Published:** 2026-05-10

**Authors:** Carlos G. Moreira, Adrian Müllner, Meltem Gönel, Pascal Hofmann, Filipe Teixeira, Rosa C. Paolicelli, Inês Dias, Sergio I. Nemirovsky, Christian R. Baumann, Daniela Noain

**Affiliations:** ^1^ Department of Neurology University Hospital Zurich, University of Zurich Zurich Switzerland; ^2^ Department of Health Sciences and Technology, D‐HEST ETH Zurich Zurich Switzerland; ^3^ Department of Neurosurgery University Hospital Zurich, University of Zurich Zurich Switzerland; ^4^ Department of Biomedical Sciences University of Lausanne Lausanne Switzerland; ^5^ Institute of Biological Chemistry, School of Exact and Natural Sciences (IQUIBICEN). CONICET – University of Buenos Aires Buenos Aires Argentina; ^6^ University Center of Competence Sleep and Health Zurich (CRPP), University of Zurich Zurich Switzerland; ^7^ Neuroscience Center Zurich (ZNZ) Zurich Switzerland

## Abstract

**Objective:**

Traumatic brain injury (TBI) significantly reduces the quality of life for millions of survivors worldwide, causing persistent brain tissue damage and cognitive impairments, with no established therapeutic interventions currently available. Slow‐wave activity, a hallmark of deep sleep, has been implicated in recovery after TBI, but pharmacological approaches to enhance it lack specificity and scalability, complicating efforts to identify slow‐wave activity as a direct mechanistic contributor and severely limiting clinical translation.

**Methods:**

To overcome these limitations, we developed a preclinical closed‐loop auditory stimulation (CLAS) paradigm that targets sleep's slow waves, enabling highly specific and temporally precise enhancement of slow‐wave activity. Therefore, we delivered 30‐ms sound triggers targeting the up‐phase of real‐time detected slow‐waves (upCLAS: TBI n = 8), or no sound stimulation (mockCLAS: non‐TBI n = 8, TBI n = 7) during sleep to healthy controls (non‐TBI) or brain injured (TBI) rats. Concomitantly, we assessed the ability of upCLAS‐enhanced sleep to counteract brain tissue damage (primary outcome) and symptomatic sequelae (secondary outcome) of TBI.

**Results:**

Bayesian analysis revealed that sound‐mediated slow‐wave activity enhancement: (1) reduces diffuse axonal injury, with TBI mockCLAS posterior estimates falling outside the 95% confidence intervals of both other groups, whereas the posterior distributions of TBI upCLAS and non‐TBI groups largely overlapped (~13% posterior differences >0), consistent with a negligible effect size between groups; (2) decreases demyelination, with approximately 97% posterior differences >0 between TBI mockCLAS and TBI upCLAS groups, compared to approximately 60% between non‐TBI and TBI upCLAS groups; and (3) preserves cognitive ability, with recognition indexes in the novel object recognition test significantly above chance level in non‐TBI (*p = 0.031) and TBI upCLAS (*p = 0.026) groups, in contrast to mockCLAS‐treated TBI rats (*p* = 0.156), presenting pronounced cognitive deficit. Furthermore, microglial response to brain injury was increased by deep sleep enhancement, with reduced ionized calcium‐binding adaptor molecule 1+ area coverage in TBI upCLAS rats (*p = 0.0445) compared to non‐TBI ones.

**Interpretation:**

These results unambiguously demonstrate slow‐wave activity enhancement confers robust disease modification following TBI while overcoming major limitations of other preclinical approaches. Our findings constitute proof‐of‐concept that boosted sleep intensity mitigates histopathological and cognitive sequelae of brain trauma, suggesting that a clinically relevant, nonobtrusive, sleep‐based therapy may represent a novel therapeutic intervention for TBI survivors. ANN NEUROL 2026;100:242–254

Every year, 69 million people worldwide are estimated to sustain traumatic brain injury (TBI), and nearly half of these individuals report at least 3 persistent post‐traumatic symptoms, including cognitive deficits.^1^, ^2^, ^3^ One common TBI‐associated neuropathology is diffuse axonal injury (DAI). Damage incurred by brain white matter from trauma forces causes complex cytoskeletal changes that lead to rapid accumulation of proteinaceous products, such as amyloid precursor protein (APP), in axonal swellings.^4^ In severe cases, DAI can result in demyelination, ultimately ending in axotomy and cell death,^5^ leading to significant functional impairments in TBI patients. As a first response to mitigate brain damage post‐TBI, acute recruitment of microglia—the central nervous system resident innate immune cells—plays a critical role, detecting and rapidly responding to tissue injury and often acting to remove cellular debris.^6^, ^7^ Defective microglia response in TBI may be predictive of cognitive deficits.^8^

It is known that deep sleep supports the accumulation and removal of toxic proteinaceous metabolites from the rodent brain via regulation of release and clearance processes, which may perhaps explain the impact of sleep modulation onto soluble protein content.^9^, ^10^, ^11^, ^12^ However, whether such sleep‐regulated pathways mediate the accumulation/removal of larger protein complexes and/or cellular debris after TBI is less certain, suggesting that other processes downstream of sleep may be relevant. Glia neuroinflammatory response, on the other hand, has been shown to play a positive role in the acute phase after trauma,^13^, ^14^ indicating that microglia could initially mediate neuroprotective processes after TBI. Strikingly, direct links between glial cells and processes driving the sleep–wake cycle have been identified recently,^15^, ^16^, ^17^ enabling the notion that modulation of sleep/circadian cycle could alter microglial responses in the context of disease.

Therefore, by interacting with potentially neuroprotective brain processes triggered on tissue damage, sleep modulation has been conceptualized as a potential therapy after TBI.^18^, ^19^, ^20^ We have shown that pharmacologically increased slow wave activity (SWA) during the first days after rodent TBI significantly reduces APP accumulation and preserves post‐traumatic cognitive ability.^21^ However, pharmacological approaches to enhance SWA alter the cyclic transitions between sleep stages and physiological non‐rapid eye movement sleep (NREMS),^22^, ^23^ therefore, do not constitute a sleep architecture‐preserving methodology. In addition, sleep pharmacotherapy lacks the specificity and innocuousness required for scaled clinical implementation. Instead, an ideal therapeutic strategy for TBI victims should preserve sleep architecture, therefore avoiding further negative effects over memory‐linked rapid eye movement (REM) sleep (REMS),^24^ and enhance SWA via a nonpharmacological, noninvasive method, such as up‐phase‐targeted closed‐loop auditory stimulation (upCLAS) of sleep slow waves,^25^ which has shown promising home‐based implementation in healthy humans^26^ and patient cohorts.^27^

In this proof‐of‐concept study, we delivered upCLAS to rats immediately after TBI to: (1) determine the efficacy, safety, specificity, and stability of upCLAS as SWA modulator after TBI; (2) demonstrate the critical role of increased SWA in reducing TBI‐associated neuropathology and post‐traumatic cognitive deficits; and (3) explore the extent of a potential sleep‐mediated cellular immune response in the brain after TBI.

## Materials and Methods

### Experimental Design

We performed the experiment in multiple batches of electroencephalography (EEG)/electromyography (EMG) implanted rats, allocated evenly across 3 experimental groups: non‐TBI rats (n = 8) underwent no TBI and post‐traumatic mock closed‐loop auditory stimulation (CLAS) of slow waves (events flagged but no sound delivered); TBI mockCLAS rats (n = 7) underwent TBI and post‐traumatic mock CLAS of slow waves; and TBI upCLAS rats (n = 8) received both TBI and up‐phase‐targeted CLAS of slow waves (pink noise triggers delivered to the slow waves' up‐phase). Following the first novel‐object recognition test (NORT) (Supporting Information), we transferred the rats to individual custom‐made acrylic‐glass cages (26.5 × 42.5 × 43.5 cm). Each cage was positioned inside a sound‐attenuated chamber for 24‐hour undisturbed EEG/EMG baseline (BL) recording (see Supporting Information).^25^ The day after, rats underwent closed‐skull TBI induction or sham surgery (see Supporting Information). On the following day, we initiated a 5‐day auditory stimulation protocol.^25^ CLAS was stopped at the end of the 5th day, and the animals were recorded for 2 more days without stimulation, to assess potential carry‐over effects. Duration of treatment was determined by our previous studies,^28^ in which we detected a cognitive deficit at 7 days post‐TBI, leaving a possible window for interventions of 5 days. Two weeks following sham or TBI induction, the rats were again tested in the NORT, and 28 days after sham or TBI induction, we euthanized the rats and harvested their brains for histopathological determinations (see Supporting Information). A minimum of 4 and a maximum of 7 animals per group were analyzed for each measure, as reported in detail in the figure legends. Exclusions of data in the different outcomes were related to low EEG/EMG signal quality, data loss because of system crashes, non‐engaging animal in the behavioral task (median split of NORT data) (see Supporting Information), and lack of tissue integrity or availability. All in vivo procedures and experimental interventions were performed by an unblinded experimenter, whereas all data post‐processing and analysis was executed in a double‐blinded fashion. All procedures were approved by the Veterinary Office of the Canton Zurich (license ZH231/2015) and conducted in accordance with national and institutional regulations for care and use of laboratory animals.

### CLAS of Sleep Slow Waves

We divided the animals into 2 phase‐targeted stimulation approaches: up‐phase stimulation targeting 65° and mock stimulation arbitrarily flagging slow waves at 90° with no delivery of sound (see Fig S1B). A sound trigger was delivered within 8ms (RX8 MULTI‐I/O processor, Tucker‐Davis Technologies, FL, USA) on validation of truth‐value for NREM ratio, EMG power, and phase target criteria. Non‐arousing stimuli consisted of pink 1/f‐noise clicks (30ms duration, 35 dB SPL, 2ms rising and falling slopes) whose waveforms were randomly generated in real time using the RX8 DSP routines (RPvdsEx, TDT). Pink noise was produced algorithmically by filtering broadband noise within the DSP environment to achieve a 1/f spectral profile. A fixed algorithmic configuration was used across animals to ensure stable spectral characteristics and consistent amplitude across triggers and sessions. The calibrated MF1 multi‐field magnetic speakers (TDT) mounted 50cm above the center of the chamber delivered the sound in free‐field conditions. The protocol for the mock condition was similar to up‐phase stimulation, but the sound was muted. All triggers were time‐flagged for offline analysis, including daily amount and entrainment (see Fig S1C, D). Importantly, train length was not determined by an upper amplitude threshold nor terminated by exceeding a predefined slow‐wave amplitude range. Trains of stimulated slow‐waves were defined as consecutive triggers occurring ≤1 second apart, reflecting sustained sequences of slow waves that continuously fulfilled NREM ratio, EMG, and phase‐target criteria. Additionally, throughout all stimulation days, synchronized video monitoring was used to actively inspect and confirm the absence of overt motor responses or potential stimulation‐evoked behavioral arousals. All EEG/EMG recordings were subjected to offline scoring and epochs showing visible arousals or movement artefacts were identified and excluded from subsequent analyses.

### Histopathological Determinations

DAI, as evidenced by APP immunoreactive axonal accumulation,^29^ is a hallmark of post‐traumatic neuropathological sequelae. Concomitantly, myelin is damaged through both direct axonal injury and indirect secondary injuries, such as DAI, astroglial damage, autoimmunity, and cell body injury in TBI.^30^ To assess whether CLAS contributes to preventing DAI and further pathophysiological mechanisms downstream of it, we performed APP staining and stereological quantification of axonal bulbs, as well as assessed myelin integrity of axonal projections through myelin basic protein (MBP) staining followed by signal density analysis in the corpus callosum along standardized protocols (see Supporting Information).

### Microglia Profiling

#### Immunofluorescence

Brain slices were permeabilized with 0.5% Triton X‐100 (Sigma‐Merck, X100, MO, USA) in phosphate‐buffered saline (PBS) for 1.5 hours at RT and blocking was performed with 2% bovine serum albumin (VWR, 9048‐46‐8, PA, USA) in permeabilization buffer (1 hour at RT). Primary antibody against ionized calcium‐binding adaptor molecule 1 (IBA1, 1:1000, FUJIFILM Wako, 019–19,741, VA, USA) was diluted in blocking buffer and slices were incubated overnight at 4°C. After 3 washes with PBS, samples were incubated with fluorescently labelled secondary antibodies (Alexa Fluor Plus, Thermo Fisher Scientific A32790, A32794, MA, USA), diluted 1:1000 in blocking buffer, for 2 hours at RT. After 3 PBS washes, nuclei were stained for 20 minutes at RT with 1μg/ml 4′,6‐diamidino‐2‐phenylindole (DAPI) in PBS. Thermo Fisher Scientific, D1306, MA, USA. Mowiol 4–88 (Sigma‐Aldrich, 81381, MO, USA) was used as mounting medium and the slides dried overnight before the acquisition.

#### Confocal Microscopy and Imaging Analysis

Confocal microscopy was performed by using Stellaris 5 confocal laser scanning system (Leica Microsystems, Wetzlar, Germany), using a dry 20× or immersion 63× objective. Scale bars are reported in the figure legends. Confocal acquisitions were processed using ImageJ Software (National Institutes of Health, MD, USA) or Imaris Software (Bitplane, Zurich, Switzerland). Microglial density was measured on 20× magnification z‐stacks acquired from the somatosensory cortex immediately adjacent to the corpus callosum area where anti‐APP and anti‐MBP signals were quantified, and calculated based on co‐localization of IBA1 and DAPI signals. DAPI and IBA1 signals were thresholded using fixed settings within the same experiment. DAPI signal was multiplied by IBA1 signal per each focal plane of the z‐stack acquisition, using the “image calculator” function. The resulting mask was max‐projected and the identified microglia nuclei were counted with the “analyze particle” function. Three dimensional (3D) reconstruction was performed using Imaris Software (Bitplane), with the built‐in surface module on confocal z‐stacks acquired at 63× magnification. IBA1 volume was quantified by applying 3D surface rendering of confocal stacks, using identical settings (fix thresholds of intensity and voxel) within each experiment. Surface and volume parameters were extracted for each individual reconstructed cell.

### Statistical Analysis

We present all data as mean ± standard error of the mean (SEM) or median in violin plots. We performed statistical analyses using GraphPad Prism 8.0 (MA, USA), SPSS Statistics 26.0 (IBM, NY, USA), MATLAB R2022b (Natick, MA, USA), and R (version 4.3, Vienna, Austria). Details on statistical strategies for single parameters can be found in Supporting Information.

## Results

### SWA Enhancement on upCLAS Is Linked to Preserved Memory in TBI Rats

**FIGURE 1:**
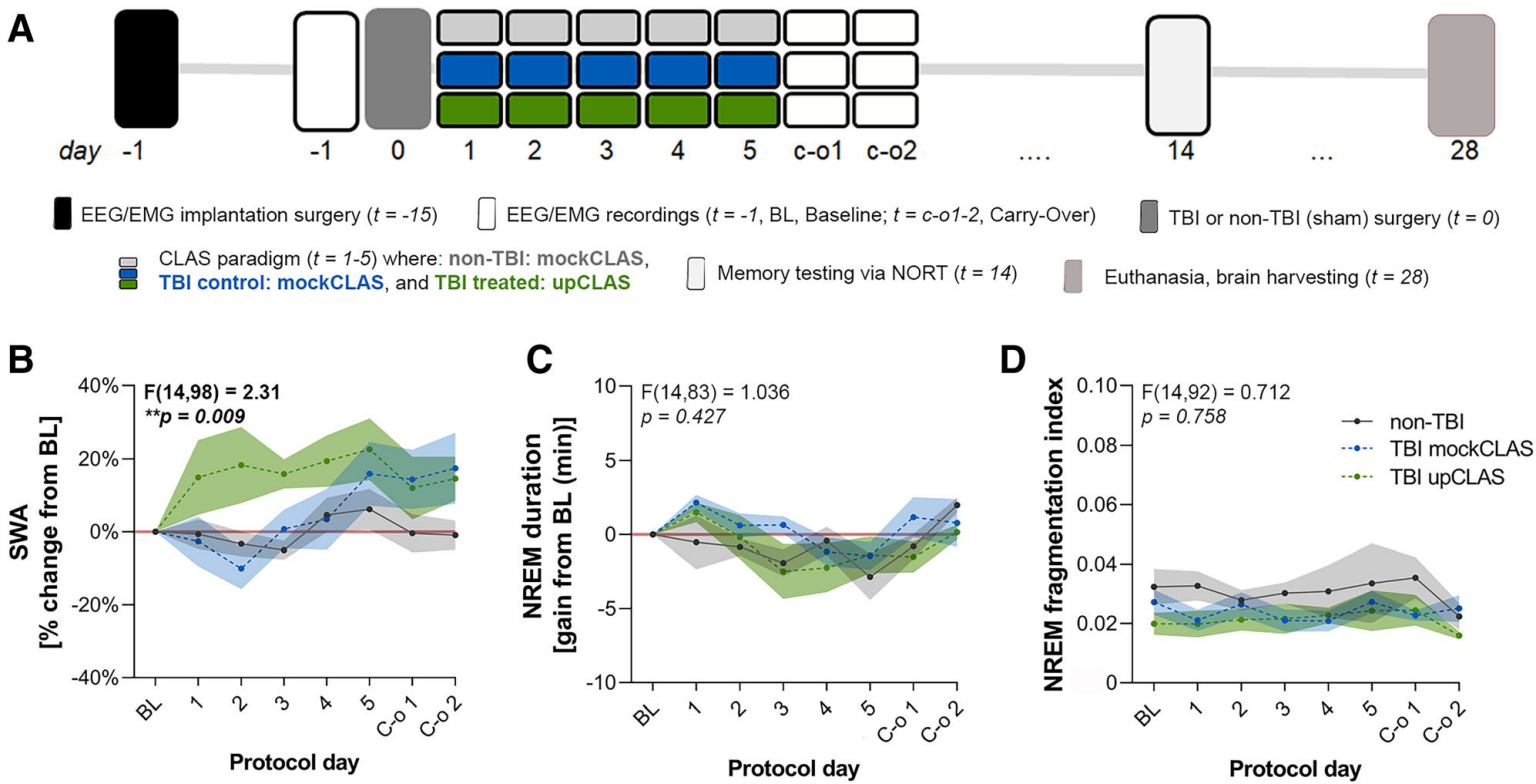
UpCLAS boosts SWA without influencing NREM sleep architecture. (A) Experimental design depicting the succession of events per protocol day for all animals in each group. Non‐TBI and TBI mockCLAS groups received no sound stimulation during the CLAS paradigm, but the corresponding stimulation events—targeted to the down‐phase—were flagged, whereas the TBI upCLAS groups received sound triggers targeted to the up‐phase of slow waves during NREM sleep. On days 9 and 23 after CLAS discontinuation, rats' memory performance was tested in the novel object recognition test (NORT) or euthanized, respectively. (B) Percentage of target phase distribution relative to total targets for each group, demonstrating target accuracy for the up‐phase of slow waves in the TBI upCLAS group. (C) SWA (2‐way analysis of variance [ANOVA], treatment × TBI interaction; *F*(14, 98) = 2.31, **p = 0.009), (C) NREM sleep duration (2‐way ANOVA, treatment × TBI interaction; *F*(14, 83) = 1.036, *p* = 0.427) and (D) NREM sleep fragmentation index (2‐way ANOVA, treatment × TBI interaction; *F*(14, 92) = 0.712, *p* = 0.758) at baseline (BL), during the 5 days of modulation,^1^, ^2^, ^3^, ^4^, ^5^ and the 2 subsequent observation days assessing carry‐over effects (C‐o 1, C‐o 2) in non‐TBI (n = 6), TBI mockCLAS (n = 7), and TBI upCLAS (n = 6) rats. mockCLAS = flagging of triggers' targets without sound delivery; non‐TBI = sham operated rats; NREM = non‐rapid eye movement sleep; SWA = slow‐wave activity; TBI = traumatic brain injury; upCLAS = up‐phase targeted closed‐loop auditory stimulation. [Color figure can be viewed at www.annalsofneurology.org]

Rats were subjected to either mock CLAS (non‐TBI, TBI mockCLAS) or upCLAS (TBI upCLAS) for 5 days acutely after induction of TBI or sham (non‐TBI) procedures, followed by 2 days of carry‐over (no stimulation) EEG/EMG recording days (Fig 1A–D). Diagnostics data show that the vast majority of upCLAS‐sound delivery successfully targeted the up‐phase (0–90° targets) of ongoing slow waves during NREMS (see Fig S1B), corroborating the accuracy of the methodology in this disease model. Our results additionally demonstrate that applying upCLAS to rats for 5 days immediately after TBI enhances overall SWA by in average 20.7 ± 4.9% in stimulation days 1 to 5 in respect to BL and elicits a temporal reorganization of slow‐waves, as evidenced by indifferent number of triggers per group with concomitant increased triggers' trains length during stimulation days (see Fig S1C, D). These changes are accompanied by no alterations in the duration or fragmentation of NREMS. Moreover, we do not observe SWA rebounds on discontinuing upCLAS. Regarding REMS, no significant differences were observed in total time or daily fragmentation index per day across groups, indicating that CLAS did not measurably alter macrostructural REMS architecture during the stimulation period.

**FIGURE 2:**
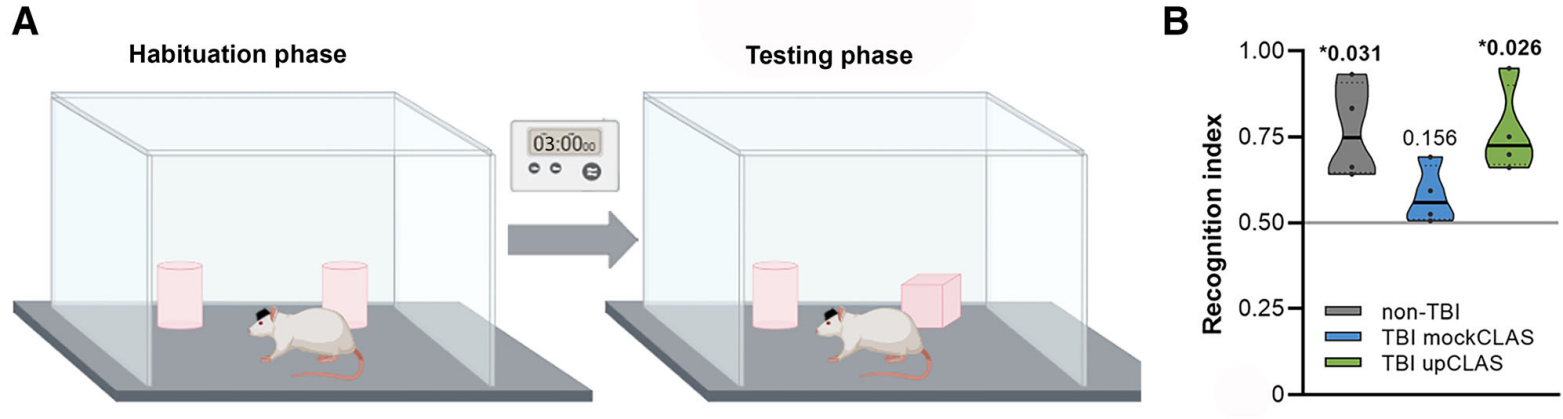
UpCLAS prevents cognitive decline in TBI animals. (A) Novel object recognition test was used to probe non‐TBI or mock/upCLAS‐treated TBI rats' cognitive performance 14 days after trauma induction. (B) Non‐TBI rats perform significantly above chance level (n = 4, non‐TBI vs recognition index at chance level = 0.5, *p = 0.031) in the novel object recognition test, in contrast to mockCLAS‐treated TBI rats (n = 4, TBI mockCLAS vs recognition index at chance level = 0.5, *p* = 0.156), which showed a pronounced cognitive deficit 14 days post‐TBI. TBI rats that received the upCLAS regime for 5 days acutely after trauma present above‐chance‐level recognition indices (n = 4, TBI upCLAS vs recognition index at chance level = 0.5, *p = 0.026) 14 days after TBI. All groups were analyzed by 1‐sample *t* test in comparison with 0.50, defined as the chance performance value equivalent to “no recognition”. mockCLAS = flagging of triggers' targets without sound delivery; non‐TBI = sham operated rats; TBI = traumatic brain injury; upCLAS = up‐phase targeted closed‐loop auditory stimulation. [Color figure can be viewed at www.annalsofneurology.org]

Nine days after CLAS paradigm discontinuation (ie, 14 days after trauma) we assessed whether delivery of upCLAS immediately after trauma alleviated sub‐chronic post‐traumatic cognitive impairment by comparing the performance of non‐TBI rats and mockCLAS‐treated TBI rats with that of upCLAS‐treated TBI rats on the novel object recognition test (Fig 2A, recognition index = 0.5, reflecting equal exploration of both objects). As expected, non‐TBI rats perform well at discriminating the novel object above chance level (1‐sample *t* test vs 0.5: *p = 0.031), whereas mockCLAS‐treated TBI animals do not (*p* = 0.156), reflecting impaired episodic memory. Rats treated with upCLAS immediately after TBI exhibit cognitive ability broadly equivalent to that of non‐TBI rats (*p = 0.026) (Fig 2B).

### upCLAS Prevents Histopathological Damage in TBI Rats

#### Reduced APP Accumulation into Axonal Bulbs

**FIGURE 3:**
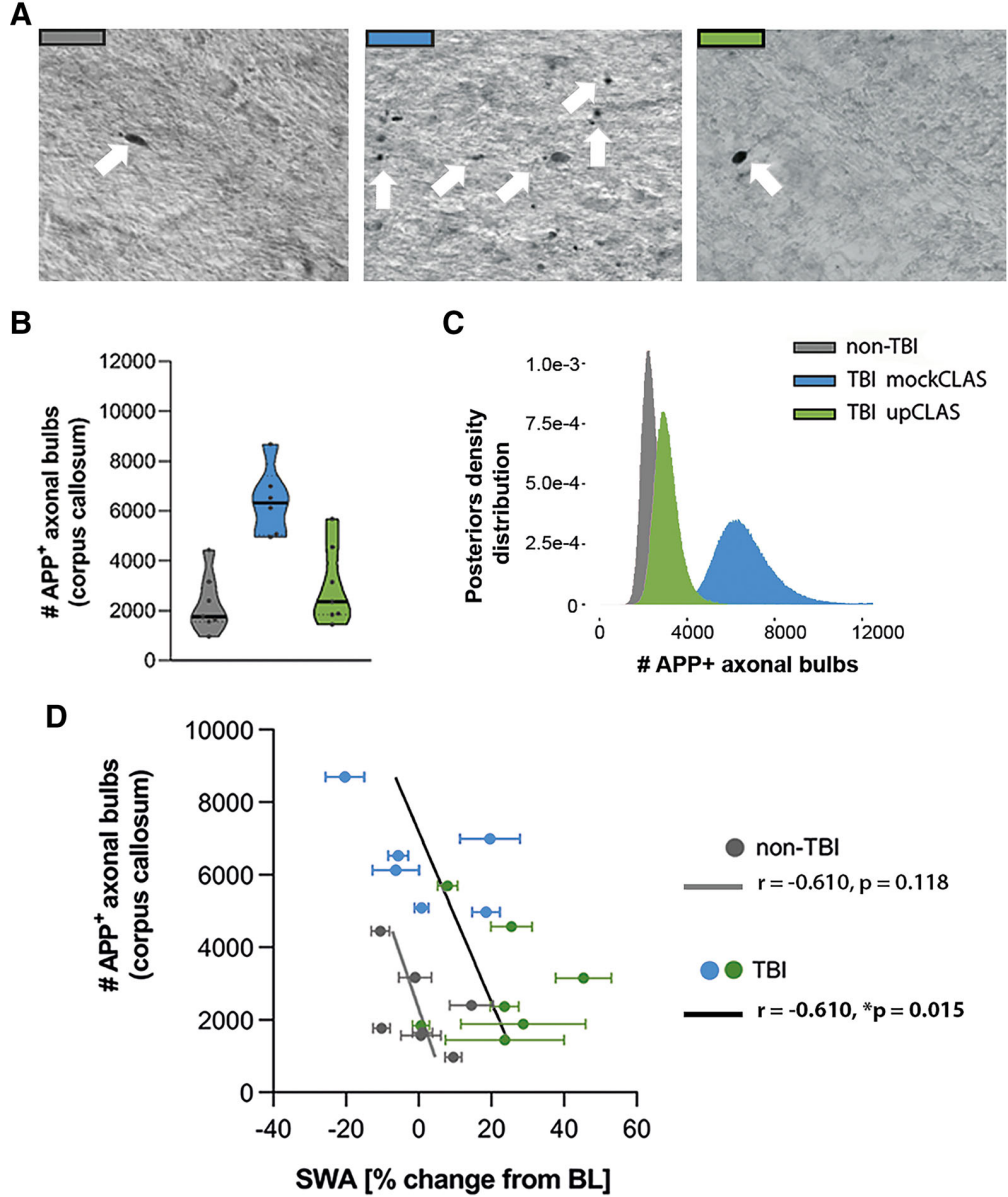
Reduced diffuse axonal damage in corpus callosum in TBI rats treated with upCLAS. (A) Micrographs at 63× magnification display low or high burden of APP+ axonal bulbs (white *arrows*) in the corpus callosum of non‐TBI (n = 7, gray), TBI mockCLAS (n = 6, blue), and TBI upCLAS (n = 7, green) rats coronal brain sections. (B) Stereological estimates of the number APP+ axonal bulbs in corpus callosum of non‐TBI, TBI mockCLAS, and TBI upCLAS rats were plotted into violins and analyzed by fitting them to a negative binomial distribution with treatment as categorical factor using a Bayesian general linear regression approach. The prior for this model was established with the coefficients of a Poisson distribution and following a student distribution. (C) Histogram of the posterior density distributions for the parameters of each treatment. Strong differentiation of the TBI mockCLAS group, whose intervals stand clearly outside the 95% CI of both other groups. The TBI upCLAS and non‐TBI posterior distributions have large superimposition with 13.04% of the posterior differences >0 (non‐TBI−TBI upCLAS). (D) Spearman's correlation indicates a negative relationship between SWA (% of change from BL) and number of APP+ bulbs in the corpus callosum of TBI animals (r = 0.610, *p = 0.015), whereas no correlation between these variables is observed in the non‐TBI group. APP = amyloid precursor protein; BL = baseline; CI = confidence interval; mockCLAS = flagging of triggers' targets without sound delivery; non‐TBI = sham operated rats; OD = optical density; SWA = slow‐wave activity; TBI = traumatic brain injury; upCLAS = up‐phase targeted closed‐loop auditory stimulation. [Color figure can be viewed at www.annalsofneurology.org]

In DAI, APP‐filled bulbs form in damaged axons disrupting critical cortical–subcortical pathways, leading to widespread cognitive dysfunction and other neurological sequelae in both TBI patients and animal models.^31^, ^32^ Therefore, preserved DAI may explain spared behavioral performance in upCLAS‐treated TBI rats. To determine the extent of post‐traumatic DAI rescue promoted by nonpharmacologically modulated SWA, we performed APP‐specific immunostainings (Fig 3A–D) followed by stereological quantification of APP+ axonal bulbs in the anterior portion of the corpus callosum, a particularly vulnerable white tract bundle, on coronal slices from brains harvested 23 days after CLAS paradigm discontinuation (ie, 28 days after trauma). Comparison of Bayesian posteriors distribution showed substantially higher APP+ axonal bulb estimates for white matter tracts in the brains of mockCLAS‐treated TBI rats than in non‐TBI and upCLAS‐treated TBI rats' brains, demonstrating for the first time that sound enhanced SWA shortly after trauma leads to reduced post‐traumatic secondary brain injury. Moreover, number of APP+ axonal bulbs was positively correlated with stimulation‐mediated changes in SWA measures.

#### Preserved Levels of MBP in Axonal Tracts

**FIGURE 4:**
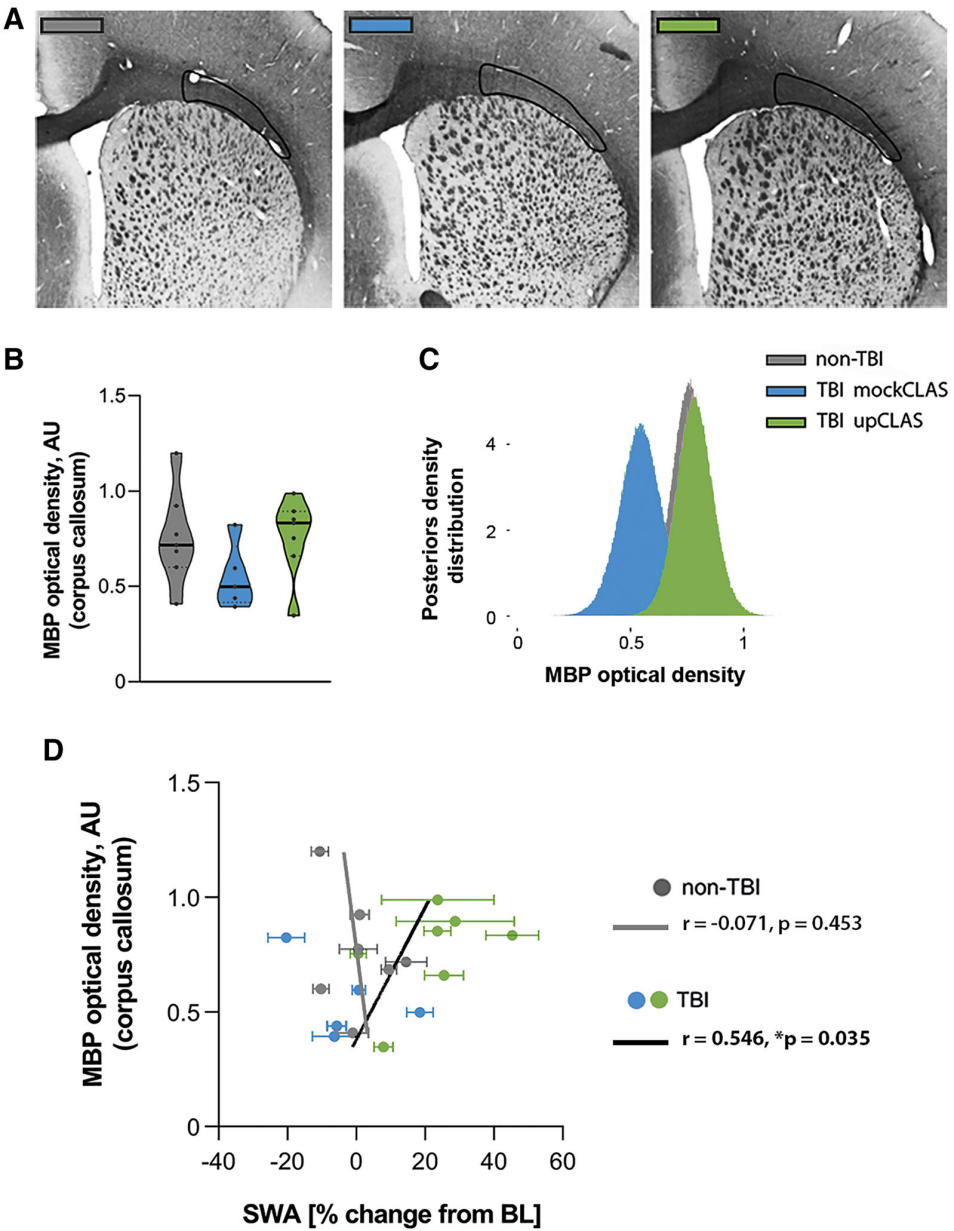
Reduced corpus callosum demyelination in TBI rats treated with upCLAS. (A) Micrographs at 10× magnification displaying representative MBP staining levels in the corpus callosum of non‐TBI (n = 7, gray), TBI mockCLAS (n = 5, blue), and TBI upCLAS (n = 7, green) rats coronal brain sections. (B) OD values (AU) in the corpus callosum of each group were plotted into violins and analyzed by a Bayesian linear regression fitted to robust priors (student_t in brms) for each group set according to their approximate normal parameters. (C) Histogram of the posteriors' density distribution for the parameters of each treatment. Comparisons showed an estimated difference in OD between TBI mockCLAS and TBI upCLAS of 0.24 ± 0.127 (AU), with 97.1% of the posterior differences >0, compared to 60.4% when between non‐TBI and TBI upCLAS groups. (D) Spearman's correlation indicates a positive relationship between SWA (% of change from BL) and MBP OD (AU) in the corpus callosum of TBI animals (r = 0.546, *p = 0.035), whereas no correlation between these variables is observed in the non‐TBI group. AU = arbitrary units; BL = baseline; CI = confidence interval; MBP = myelin basic protein; mockCLAS = flagging of triggers' targets without sound delivery; non‐TBI = sham operated rats; OD = optical density; SWA = slow‐wave activity; TBI = traumatic brain injury; upCLAS = up‐phase targeted closed‐loop auditory stimulation. [Color figure can be viewed at www.annalsofneurology.org]

Furthermore, DAI‐triggered demyelination represents one of the critical TBI‐triggered secondary pathways to axotomy, eventual cell death, and consequent functional impairment.^33^ Therefore, we also assessed the extent of post‐traumatic demyelination with specific immunostaining of the MBP (Fig 4A–D), involved in myelin sheath formation and used in clinical settings to evaluate the severity of TBI.^34^ We found that the corpus callosum of mockCLAS‐treated TBI rats exhibited lower MBP staining levels than non‐TBI controls as assessed by optical density quantification, highlighting the damaging effect of mild TBI on principal fiber bundles in the rat brain. In contrast, the corpus callosum of upCLAS‐treated TBI rats show close‐to‐healthy MBP staining levels, with optical density values significantly overlapping (95% confidence interval) with the non‐TBI group. Last, MBP optical density values positively correlate with stimulation‐mediated changes in SWA measures.

### Enhanced Microglial Response on upCLAS in TBI Rats

#### Supported Microglial Response in Sleep‐Enhanced TBI Brains

**FIGURE 5:**
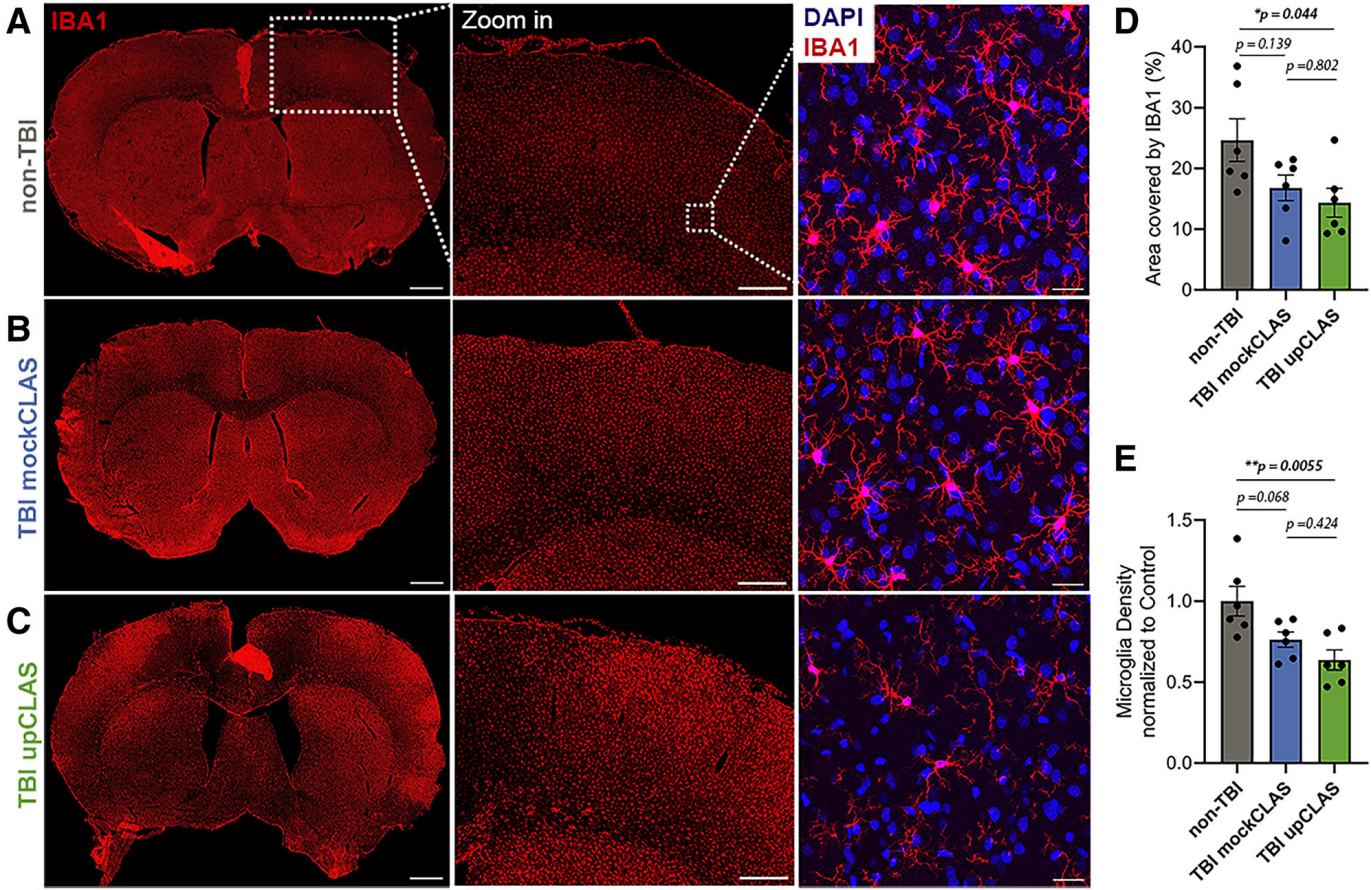
Clustered microglia distribution in upCLAS‐treated TBI brains. (A) Representative photomicrographs of IBA1 immunosfluorescence of 40μm coronal brain sections from non‐TBI rats (n = 6), (B) TBI rats treated with mockCLAS (*n* = 6), and (C) TBI rats treated with upCLAS (n = 6) for 5 days on injury. Distinct distributions are observed across the groups, as also observed in the zoomed in panels. High magnification shows IBA1+ microglia (red) and DAPI staining cell nuclei (blue). (D) Quantification of cortical areas covered by IBA1+ cells in all 3 groups, shows reduced coverage in the TBI upCLAS animals (1‐way analysis of variance [ANOVA], non‐TBI vs TBI upCLAS *p = 0.044), evidencing a non‐physiological distribution of microglia (reactive profile). (E) Microglia density analysis also revealed a significantly lower number of IBA1+ cells/μm^3^ in upCLAS‐treated TBI rat brains, characteristic of a patched distribution pattern. Bars represent 20μm. IBA1 = ionized calcium‐binding adapter molecule 1; DAPI = 4′,6‐diamidino‐2‐phenylindole; mockCLAS = flagging of triggers' targets without sound delivery; non‐TBI = sham operated rats; TBI = traumatic brain injury; upCLAS = up‐phase targeted closed‐loop auditory stimulation. [Color figure can be viewed at www.annalsofneurology.org]

Acute microglial reactivity after TBI may benefit the brain healing process by facilitating waste removal and clearance of cellular debris. Concomitantly, glia function has been linked to the sleep/wake cycle.^15^, ^17^ To investigate whether CLAS‐mediated SWA enhancement in the post‐traumatic acute phase triggered a differential microglial response in rat TBI brains that may potentially underlie the observed long‐term ameliorated APP and MBP profiles, we explored IBA1 (microglia marker) fluorescent staining distribution in coronal non‐TBI, TBI mockCLAS, and TBI upCLAS brain sections 28 days after trauma. We found a visually distinct IBA1 distribution profile between the groups, with non‐TBI brain presenting a homogenous scattering typical of homeostatic microglia (Fig 5A–E). TBI mockCLAS brains, on the other hand, showed an intermediate distribution profile. In contrast, TBI upCLAS brains exhibited IBA1 fluorescence clustered in localized areas of the brain, particularly noticeable in cortical structures. We corroborated the visual observations via quantifications of microglia density and area covered by IBA1 specifically in the cortical area immediately adjacent to the corpus callosum region where DAI was quantified. This analysis demonstrated statistically significant differences between non‐TBI and TBI upCLAS brains (1‐way analysis of variance [ANOVA], density: **p = 0.0055; area covered: *p = 0.044), but only observing non‐significant trends between non‐TBI and TBI mockCLAS groups.

#### Distinct Microglial Morphology in Sleep‐Enhanced TBI Rats

**FIGURE 6:**
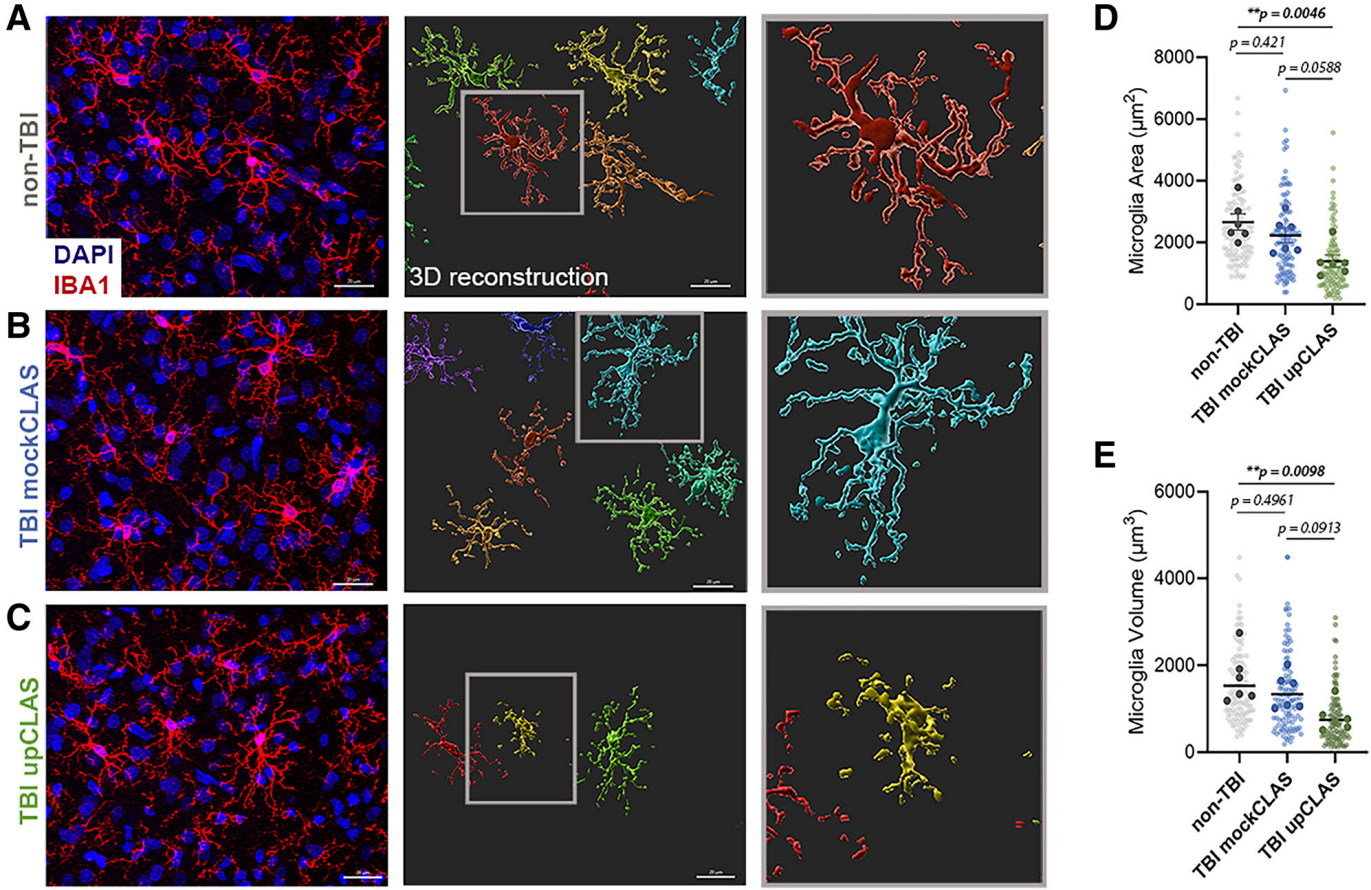
Responsive microglia morphology in upCLAS‐treated TBI brains. (A) Representative photomicrographs (left), Imaris‐3D profile reconstructions (middle), and detailed representative 3D insets (right) of immunofluorescence‐stained IBA1+ cells in somatosensory cortical regions of non‐TBI (n = 122 total cells), (B) TBI mockCLAS (n = 117 total cells), and (C) TBI upCLAS (n = 109 total cells) brains demonstrate differences between upCLAS‐treated group and the others. (D) 3D‐reconstruction derived quantification of cellular area and (E) volume in all 3 groups confirm qualitative observations, demonstrating that TBI upCLAS animals present significantly lower values in both measures than non‐TBI rats (1‐way analysis of variance [ANOVA], Tukey's multiple comparisons test: non‐TBI vs TBI upCLAS, area: **p = 0.0046; volume: **p = 0.0098), indicating a more responsive microglia profile in SWA‐enhanced rat brains. Trends were observed toward lower area and volume values in TBI upCLAS group compared to TBI mockCLAS (1‐way ANOVA, Tukey's multiple comparisons test: TBI mockCLAS vs TBI upCLAS, area: *p* = 0.0588; volume: *p* = 0.0913), suggesting TBI mockCLAS brain to present intermediate values between non‐TBI and TBI upCLAS groups. Bars represent 20μm. mockCLAS = flagging of triggers' targets without sound delivery; non‐TBI = sham operated rats; TBI = traumatic brain injury; upCLAS = up‐phase targeted closed‐loop auditory stimulation; 3D = tridimensional. [Color figure can be viewed at www.annalsofneurology.org]

Moreover, visual inspection of high magnification images (see Fig 5A–C, blue: DAPI; red: IBA1) from somatosensory cortical regions indicated that microglia morphology in the TBI upCLAS brains appear divergent to that of the other groups, with less processes and smaller size. To confirm these qualitative observations, we additionally performed microglia morphological analyses through 3D cellular reconstruction from high magnification micrographs of between 109 and 122 IBA1^+^ cells from n = 6 subjects per group (Fig 6A–E) and compared derived quantitative measures of cellular area and volume. We did not find significant differences in microglia area or volume between non‐TBI and TBI mockCLAS brains, whereas microglia morphology in TBI upCLAS brain tissue was significantly different from that of non‐TBI animals (1‐way ANOVA, area: **p = 0.0046; volume: **p = 0.0098), with smaller area and volume values suggesting a reactive profile.

## Discussion

The present results demonstrate that upCLAS effectively enhances SWA, reduces DAI, preserves myelin integrity, and mitigates post‐traumatic memory impairment in TBI rats, along with apparently increased microglia reactivity. To our knowledge, this is the first report demonstrating a global beneficial effect of a nonpharmacological, clinically scalable sleep‐based treatment on structural brain integrity following TBI, together with first insights into a potential underlying mechanism.

Multiple studies in humans and animals have investigated the effects of CLAS on boosting^35^, ^36^ and disrupting^37^, ^38^ SWA. To date, however, no report has evidenced the effectiveness of CLAS in the context of brain disease. Here, we demonstrate the efficacy of CLAS in rodent TBI by showing upCLAS is technically feasible and reproducible, selectively delivered during NREMS, and effective in enhancing SWA in TBI rats over multiple days, while not altering sleep duration or fragmentation. Moreover, analysis of triggers showed that upCLAS did not significantly increase the total number of daily triggers during the stimulation period, but rather progressively shifted the internal structure of slow‐wave sequences, increasing the proportion of long trains, potentially suggesting that its effect was not merely generating more slow‐wave events, but promoting greater temporal continuity of slow‐waves. Analysis of carry‐over effects on EEG spectral power in NREM sleep revealed a renormalization of SWA on CLAS' discontinuation. Of note, however, renormalized levels of SWA in upCLAS TBI rats do not resume to non‐TBI levels (see Fig 1A). Moreover, SWA levels in mockCLAS TBI rats appear to climb naturally up starting day 4 to 5 of the protocol. This is consistent with previous report of transiently increased SWA approximately 1 week after trauma induction in this TBI model,^39^ as well as data from others showing a similar effect in human TBI subjects, where injury severity was directly linked to higher SWA power.^40^ Therefore, the confluence of the mockCLAS TBI and upCLAS TBI SWA curves during the carry‐over days implies a complete renormalization of SWA values back to their “new” BL level on upCLAS discontinuation. The observed reversibility on discontinuation is consistent with findings reported in human CLAS^41^ and denotes a desirable high degree of reversibility. These findings open valuable new avenues for translation into clinical therapy avoiding pharmacological SWA enhancement after TBI, which is hindered by ethical and practical limitations. In addition to their dependency, tolerance, and sleep‐architecture‐altering issues, sleep‐inducing pharmacotherapies impede swift clinical monitoring of patients after TBI.

Both intracellular and extracellular mechanisms may be responsible for the reduction in APP accumulation in axonal bulbs following upCLAS‐enhanced SWA. A widely discussed possibility is that enhanced glymphatic function facilitates removal of proteinaceous products during sleep,^42^ and upregulation of protein players within other processes has been proposed, such as the ubiquitin/proteasome system.^43^ However, direct links remain to be fully established between these homeo‐/proteo‐static pathways and TBI‐triggered pathomechanisms, with focus on whether such systems are truly able to facilitate clearance of high molecular weight proteins and cellular debris. Moreover, TBI‐induced secondary injury extends beyond abnormal protein accumulation because of impaired axonal transport. For instance, TBI‐triggered proteinolysis mediated by calpain activation after axonal injury often leads to demyelination.^44^ Our finding of preserved MBP staining intensity in the corpus callosum—the main white matter tract in the rodent brain—indicates conserved myelination levels in upCLAS‐treated TBI rats and, therefore, suggests that acute auditory enhancement of sleep SWA may be necessary and sufficient to promote neuroprotection after TBI.

Spared post‐traumatic cognitive ability on sound‐mediated sleep enhancement is a compelling finding. However, these results must be interpreted with caution. The high variability of NORT scores across all experimental groups required us to perform a median‐split analysis to identify and exclude a subset of rats in each group that presented very low or no behavioral engagement in the test. Considering that a similar number of rats were excluded from each group, we assume their inactivity was not related to treatment and surmise that they were particularly susceptible to certain experimental conditions, such as environmental noise and odors. Nonetheless, our preliminary analysis of behavioral scores from rats that actively engaged in the test (see Fig 2) strongly suggests that acute enhancement of SWA helps preserve cognitive abilities after TBI.

**FIGURE 7:**
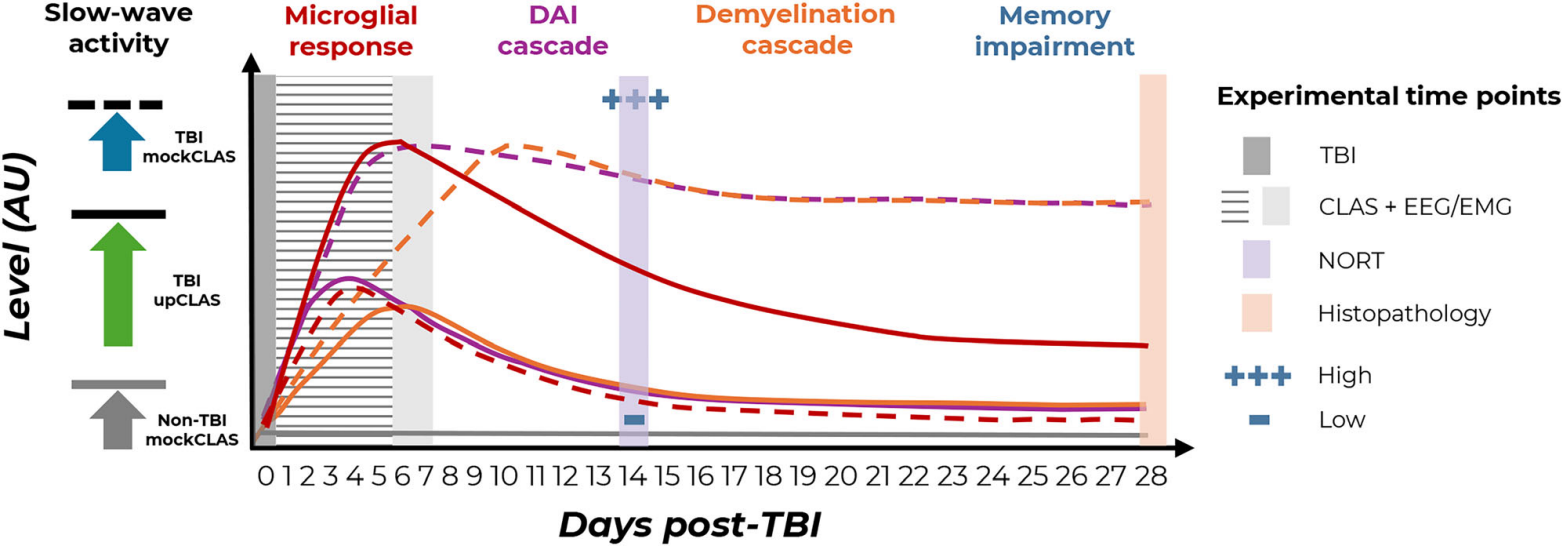
Working model on CLAS‐mediated effects after TBI. Schematic representation of a working model combining acquired data at specific time points (TBI: gray; CLAS + EEG/EMG: striped pattern + light gray; NORT: light purple; histopathology: light orange) and potential temporal dynamics of the outcome measures. Treatment responses of non‐TBI rats receiving mockCLAS (no stimulation; dark gray *arrow* and *solid line* gray curve), TBI rats receiving mockCLAS (no stimulation; blue *arrow* and *dashed line* curves), and TBI rats receiving upCLAS (up‐phase targeted CLAS, green *arrow* and *solid line* curves) during 5 consecutive days after trauma are portrayed. Non‐TBI mockCLAS rats maintain a temporally stable level for all outcome measures (solid line gray curve), whereas TBI mockCLAS rats display a robust DAI (dashed dark purple curve) and subsequent demyelination (dashed dark orange curve) responses. upCLAS‐treated TBI rats present moderate noxious responses (solid dark purple and dark orange curves) in the acute post‐traumatic phase, which resolve back to non‐TBI levels by day 28 post trauma. Such patterns of histopathological progression support the observed low memory impairment (blue–sign) at day 14 post trauma in upCLAS‐treated TBI rats, whereas mockCLAS‐treated TBI ones experience a high degree of memory impairment (blue +++ signs). Microglial response is speculated moderate in the mockCLAS‐treated TBI rats' brains, reaching levels similar to these seen in non‐TBI mockCLAS rats. On the contrary, upCLAS‐treated TBI rats experience a more robust microglial response on trauma, such as the profile is not resumed to the level of non‐stimulated animals at 28 days post trauma. Overall, the model suggests a temporally sustained neuroprotective microglial response on upCLAS that protects the brain from DAI, demyelination and consequent cognitive decline. CLAS = closed‐loop auditory stimulation; DAI = diffuse axonal injury; EEG = electroencephalography; EMG = electromyography; mockCLAS = flagging of triggers' targets without sound delivery; non‐TBI = sham operated rats; TBI = traumatic brain injury; upCLAS = up‐phase targeted closed‐loop auditory stimulation; [Color figure can be viewed at www.annalsofneurology.org]

In association with alleviated TBI outcomes at functional (day 14) and neuropathological (day 28) levels, we also observed an altered microglial profile in upCLAS‐treated brains 28 days post‐TBI, characterized by altered IBA1 reactivity and morphology. These key results suggest that enhanced SWA likely altered microglia reactivity in the acute phase after trauma, which remained elevated at 28 days post‐injury and may underpin the observed reduced histopathological post‐traumatic sequelae (Fig 7). The exact mechanisms at play remain to be determined. It has been recently shown that microglia Ca^2+^ activity is naturally higher during sleep than wakefulness^45^ and growing evidence links Ca^2+^ activity in microglia to enhanced phagocytic capacity.^46^ Therefore, it is conceivable that acute post‐TBI treatment with upCLAS propitiates phagocytic activity in the injury site, known to contribute to clearance of debris and release of neurotrophic factors that support neuronal survival and recovery.^47^ However, heightened microglial response has been also conceived as a potential harbinger of worsened TBI outcomes.^6^ Therefore, further experiments are necessary to fully understand the role of SWA enhancement in modulating microglia response and profiles and their temporal timing in TBI, including determination of cytokines levels to concretely characterize the brain's inflammatory state.

Notably, microglia have been recently postulated to be involved in hippocampal synaptic transmission in relation to regulation of sleep duration during the light/dark cycle. Microglia depletion can disrupt sleep/wake cycles and circadian rhythms,^48^ suggesting a tight interplay between this crucial cellular population of the neuro‐immune response and sleep quality/quantity. Alterations of the circadian rhythms in microglia can deeply affect the immune response, phagocytic function, metabolism, and other aspects of microglia, which play a key role in neurological diseases.^17^, ^49^ In this line, upCLAS‐mediated SWA enhancement in a mouse model of Alzheimer's disease, partially rescued their intrinsic circadian misalignment observed in the light–dark transition,^50^ suggesting an indirect role of upCLAS‐mediated SWA enhancement on circadian rhythm strengthening. Therefore, it is plausible that CLAS‐mediated SWA enhancement in TBI rats facilitated a more efficient sleep pressure dissipation, therefore, reinforcing their circadian/sleep–wake cycle, with consequent upregulation of the microglial response. However, dedicated and direct circadian activity assessments in non‐TBI and TBI rats have not been performed in this study and shall be considered as important additions in future experimental designs.

Conversely, mockCLAS‐treated TBI animals 28 days post‐injury exhibited microglial morphology and density akin to non‐TBI animals. This microglial phenotype may be attributed to a resolved acute inflammatory response and the subsequent return of this cell population to a more homeostatic state (Fig 7). Microglial response peaks shortly after injury, followed by a gradual decline in reactivity markers.^51^, ^52^ In the absence of acute SWA enhancement, the microglial population in the mockCLAS‐treated TBI group's brains may have reverted at 28 days post‐TBI to a less responsive state, characterized by a ramified morphology and BL‐like density around the injury site. Taken together, our observations suggest that acute microglia reactivity may have mitigated the post‐traumatic DAI and demyelination observed in the sub‐chronic post‐traumatic phase in upCLAS‐treated animals, reflecting a role of SWA in promoting pro‐regenerative neuroimmune mechanisms, potentially enhancing clearance activity, fostering survival and repair mechanisms. Follow‐up work shall carefully explore whether the presently observed positive effect on microglia response on upCLAS remains true in chronic TBI stages (3, 6, or 12 months) and/or in the presence of secondary stressors.

Altogether, the apparent ability of upCLAS‐mediated SWA enhancement to influence the trajectory of microglial response suggests that acute sleep‐based interventions may be key in altering histopathological and behavioral recovery after TBI. In this scenario, auditory stimulation seems to facilitate a more favorable microglial microenvironment aiding positive resolution of histopathological sequelae and consequent mitigation of long‐term TBI symptoms.^53^ Although causality between SWA enhancement and specific microglial functional states remains to be established, the present findings encourage further mechanistic investigations to elucidate the exact processes by which deep sleep enhancement regulates restoratives processes involving, perhaps not only, modulation of the type and temporal kinetics of microglial reactivity, with particular focus placed on the acute (1–7 days) and chronic phases (3–12 months) of the neuroimmune response.

### Limitations

Our TBI model, developed over a decade ago, was tested only in male rats, reflecting the standards at the time. We now recognize the absence of female data on CLAS effects in TBI as a key limitation. Future studies should evaluate CLAS in well‐validated female TBI models to better assess the generalizability of our findings. Additionally, our EEG setup was limited to 2 diametrically opposed channels per rat, equidistant from the TBI site, which precluded analysis of functional connectivity and network dynamics in response to CLAS. Moreover, our exclusive focus on NREM sleep prevented detailed assessment of REM and wake states, despite evidence that altered temporal coherence and slow‐wave accumulation during wakefulness may serve as important EEG biomarkers in human and rodent TBI.^54^ Future studies incorporating LFP and multiunit recordings with multi‐electrode arrays could provide a more comprehensive view of CLAS effects across behavioral states, capturing both surface and deep network reorganization as well as post‐traumatic changes in global and local connectivity. Furthermore, the absence of circadian activity recordings limits our ability to link SWA, sleep–wake patterns, and circadian rhythms. Future studies should examine these processes jointly, because they may be altered after TBI and/or upCLAS‐TBI and influence microglial profiles. Regarding timing, assessments at 28 days post‐TBI capture a relatively understudied semi‐chronic phase. However, longer follow‐up and acute histopathology would be needed to determine whether microglial changes normalize over time and to confirm the suspected early group differences, respectively. Last, limited statistical power in behavioral analyses warrants cautious interpretation. Future studies should improve testing conditions (eg, controlling odor and noise) and include a broader range of behavioral measures.

In summary, acute post‐traumatic delivery of upCLAS is the first nonpharmacological sleep‐based therapeutic tool to significantly attenuate post‐traumatic histopathological sequelae and, therefore, alleviate cognitive deficits, concomitant with a sustained adaptative microglial response. Our data encourages not only further preclinical investigations into the mechanisms governing these associations, but meticulous translation of CLAS technology into clinical environments to test its safety and efficacy in TBI patients. Ultimately, our findings offer an attractive platform for testing the effect of nonobtrusive and nonpharmacological deep sleep enhancement on clinical and radiological outcomes in TBI patients.

## Author Contributions

C.G.M., R.C.P., C.R.B., and D.N. contributed to the conception and design of the study; C.G.M., A.M., M.G., P.H., F.T., I.D., S.I.N., and D.N. contributed to the acquisition and analysis of data; C.G.M., R.C.P., and D.N. contributed to drafting the text or preparing the figures.

## Potential Conflicts of Interest

Nothing to report.

## Supporting information


**Data S1.** Supporting Information

## Data Availability

The data related to this study is included in its entirety in the report. Further requests may be directed to the corresponding author.
