# Supplementary material for: Auditory Stimulation of Slow‐Wave Sleep Promotes Recovery after Brain Injury in an Animal Model
Source: Ann Neurol. 2026 May 10;100(2):242–54. doi: 10.1002/ana.78234 (PMC13387972; doi:10.1002/ana.78234)
Supplement: Supplementary file 1 — Data S1. Supporting Information [file ANA-100-242-s001.docx]

**Supporting Information**

1. **Supplementary materials and methods**

## 1.1 Animals and husbandry

## We used 20 young-adult male Sprague-Dawley rats (Charles River, Italy) weighing 250–300g, and group-housed them in standard individually ventilated cages (T2000) prior to interventions. Following surgery, we housed the animals in pairs for a minimum of 14 days for recovery, with food and water available ad libitum, and handled them daily for postoperative monitoring, body-weight check, and familiarization with the experimenter. The animal-room temperature was maintained at 22–23 °C, and animals were kept on a 12h light–dark cycle. All procedures were approved by the Veterinary Office of the Canton Zurich (license ZH231/2015) and conducted in accordance with national and institutional regulations for care and use of laboratory animals.

## 1.2 EEG/EMG implantation surgery

## We surgically implanted electrodes in all animals for continuous recording of electroencephalography and electromyography (EEG/EMG) as described previously ^1^. Briefly, we inserted four stainless steel miniature screws (Hasler, Switzerland), one pair for each hemisphere, bilaterally into the rats’ skulls following specific stereotaxic coordinates: the anterior electrodes were implanted 3mm posterior to bregma and 2mm lateral to the midline, and the posterior electrodes 6mm posterior to bregma and 2mm lateral to the midline. For monitoring muscle tone, we inserted a pair of gold wires as EMG electrodes into the rats’ neck muscles. All electrodes were connected to stainless steel wires, further connected to a headpiece (Farnell, #M80-8540842, Switzerland), and fixed to the skull with dental cement. We performed all surgical procedures under deep anaesthesia by inhalation of isoflurane (4.5% for induction, 2.5% for maintenance) and subsequent analgesia with buprenorphine (s.c. 0.05 mg/kg).

## 1.3 TBI induction surgery

We performed TBI induction as preciously described ^1-3^. Briefly, rats were deeply anesthetized under 2.5% isoflurane and positioned on a foam platform (L × W × T: 17 × 9 × 10 cm, stiffness: 2.84 Newton/cm). Their heads were closely bordered bilaterally with two smooth wooden walls for correct positioning during trauma induction. A 0.5–0.7 cm scalp incision over the midline in the frontal head area was made just anterior to the implanted EEG/EMG headset. After exposing the skull, the impact area (2 mm anterior to bregma, over the midline) was marked and a 1 mm-thick metal plate (1 cm diameter) was placed over the exposed target area to prevent bone fractures. A 2500 g stainless steel rod of a total length of 100 cm, diameter 2 cm, with a flat pointed silicon tip (diameter 1 mm, total tip length 4 cm) was used as falling weight. It was mounted in a slide and held by a stand at an angle of 70 degrees. The metal rod was positioned precisely over the selected point of injury, elevated to a height of 25 cm, and released by pushing a button deactivating a magnet that held the metal rod in place. Non-TBI animals underwent the exact same procedures except for the injury. Subsequently, the skin was closed and disinfected. Animals returned to their home cages and were monitored continuously for at least 1 h until they had fully recovered from sedation and normal home cage behaviour was observed.

## 1.4 EEG/EMG recordings

## To verify the effect of CLAS on EEG spectra, we conducted bilateral tethered EEG/EMG recordings in differential mode for 24 h to serve as BL and throughout all the subsequent 7 days of the protocol (5 days of stimulation, days 1–5 + 2 carry-over (c-o) days, days c-o 1 and c-o 2), applying our runtime stimulation paradigm in 5 or 6 freely moving animals simultaneously. We acquired data using a multichannel neurophysiology recording system (Tucker Davis Technologies, TDT, USA). We sampled all EEG/EMG signals at 610.35 Hz, amplified them (PZ5 NeuroDigitizer preamplifier, TDT, USA) after applying an anti-aliasing low-pass filter (45% of sampling frequency), synchronously digitized them (RZ2 BIOAMP processor, TDT, USA), recorded them using SYNAPSE software (TDT, USA), and stored them locally (WS-8 workstation, TDT, USA). We filtered real-time EEG between 0.1 and 36.0 Hz (2nd order biquad filter, TDT, USA), and EMG between 5.0 and 525.0 Hz (2nd order biquad filter and 40-dB notch filter centred at 50 Hz, TDT, USA), and fed the signals to real-time detection algorithms for non-rapid eye movement (NREM) sleep staging and phase detection.

##

## 1.5 EEG/EMG scoring and post-processing

## *Scoring:* We scored all recording files using the SPINDLE online computational tool for animal sleep data ^4^. In short, European Data Format (.edf) files, consisting of 2 parietal EEG channels and 1 nuchal EMG channel, were uploaded to SPINDLE to retrieve vigilance states with 4-second epoch resolution. The algorithm classified 3 vigilance states: wakefulness, NREMS, and REMS. Additionally, unclear epochs or interfering signals were labelled as artifacts in wakefulness, NREMS, and REMS. Wakefulness was defined by high or phasic EMG activity for more than 50% of the epoch duration and low amplitude but high frequency EEG. NREMS was characterized by reduced or no EMG activity, increased EEG power in the frequency band < 4 Hz, and the presence of slow waves. REMS was defined by high theta power (6 – 9 Hz frequency band) and low muscle tone.

## *Post-processing:* Time spent in NREMS was determined as an absolute number of minutes for BL or stimulation period. For the same days, we extracted measures of global spectral responses in the delta frequency, by processing the left-hemisphere EEG signal with a custom MATLAB routine (ver. R2016b). Briefly, we removed artifacts by detecting clipping events (15 adjacent raw EEG samples of approximately 60 ms within 55 units of the amplifier maximum or minimum), followed by a 3-point moving average (to remove frequencies greater than 80 Hz). Subsequently, we applied a basic Fermi window function, f(n)=(1+e^((5-n⁄50)))^((-1)), to gradually attenuate the first and last 2 s of each signal recorded (n = 600), and resampled the EEG signal at 300 Hz. Next, we filtered the signal between 0.5 Hz and 48 Hz using low- and high-pass zero-phased equiripple FIR filters (Parks-McClellan algorithm; applied in both directions (filtfilt); order_high = 1880, order_low = 398; -6 dB (half-amplitude) cut-off: high pass = 0.28 Hz, low‑pass = 49.12 Hz). The signal was visually inspected for any regional artifacts (2-hour sliding window) not detected during automatic scoring: within scored NREMS, brief portions (< 10 sample-points at 300 Hz) of signal >± 8 × interquartile range were reconstructed by piecewise cubic spline interpolation from neighbouring points. We performed spectral analysis of consecutive 4 s epochs (FFT routine, Hamming-window, 2 s overlap, resolution of 0.25 Hz), and normalized the power estimate of each frequency bin in relation to the total spectral power (0.5 – 30 Hz). Additionally, we calculated hourly SWA (0.5–4 Hz) as the mean spectral power of equally sized NREMS-epoch bins (12 bins during light period and 6 bins during dark period), using the digital filters mentioned above (order_high = 3758 and order_low = 3861) normalized by the hourly total power (0.5–30 Hz). Hourly values were extrapolated from this curve.

## 1.6 Online NREM sleep staging and phase detection

## Parallel rule-based NREMS staging and phase detection features were run continuously alongside EEG/EMG recordings so that sound triggers were presented in real time at every instance the stimulatory truth function compounding these features was reached (see Supplementary figure, panel a). The truth-function required simultaneous validation of spectral and electromyographic for real-time NREMS staging, and slow wave up-phase-alignment and minimal amplitude criteria to privilege stimulation during consolidated NREMS. For online NREMS staging, we implemented a rule-based decision algorithm combining spectral and electromyographic thresholds. Briefly, we computed delta (0.5–4 Hz) and high-beta (20–30 Hz) bands’ root mean square (rms) on a sliding window of 1 s using an algorithm written in RPvdsEx (Real-time Processor visual design studio, TDT, USA). Once the rmsdelta/rmshigh beta ratio, hereinafter referred to as the NREMratio, crossed a threshold individually identified during the BL recording, we further compared EMG rms to a threshold, also defined during NREMS BL, to rule out movement artefacts. The NREMratio and the EMG power thresholds for online NREM staging indicative of sustained NREMS, were extracted individually and immediately after the BL recordings: 24 h EEG/EMG data was scored automatically (see Supplementary materials) and fed to a custom-written MATLAB (ver. R2022b) script. In short, a strict estimate of NREMratio threshold during NREMS was established as +1.0 SD over the mean of the NREMratio, representing the 84.1% percentile, of the NREMratio of all NREMS epochs during BL. Similarly, EMG power in NREMS was delimited to values -1.0 SD below the mean of the EMG rms values (equivalent to a threshold at 15.9% percentile) during offline-scored NREMS. The NREMratio and EMG power percentile-based thresholds were selected to restrict stimulation to high delta-to-beta ratio and low EMG epochs, low-EMG epochs and to minimize detections during quiet wakefulness and REM sleep. These values marked the transition into consolidated NREMS in specific for each subject and were introduced into a customized SYNAPSE® (TDT, USA) project for auditory stimulation. For phase-targeted auditory stimulation of slow waves, SYNAPSE® combines the online NREMS staging feature with a phase detector. In this phase detector, a runtime very narrow bandpass filter (TDT, USA) for EEG phase detection isolated the 1-Hz component for phase targeting of slow waves, approximately 1.35 Hz in rats^5^, in each subjects’ left EEG channel. A minimal amplitude threshold was applied to the filtered signal to avoid phase detection during oscillatory activity at very low voltage, thereby preventing stimulation during low-amplitude oscillations not corresponding to consolidated slow waves. We predetermined slow wave’s 0° as the rising zero crossing, 90° as the positive peak, and 270° as the slow wave’s trough. At every identified positive zero-crossing on the filtered signal, the phase detector reset to 0° and calculated any of the selected target phases from the number of samples elapsed since the zero crossing. This method offers the chance to recognize slow waves consistently across conditions independently of the target phase.

## 1.7 Novel object recognition test

## To examine declarative memory performance, we used the NORT as described previously ^2, 6, 7^. Briefly, the test consisted of two phases: habituation and testing. In the habituation phase, the animal was presented for 10 min of exploration with two equal objects; these became the familiar objects. Three hours later, the testing phase consisted of reintroducing the animal to the context and presenting both an identical copy of the familiar object, to avoid olfactory cues, and a novel object. We left the animal free to explore both objects for 5 minutes. The testing phase was acquired and analysed using dedicated software (Ethovision, Noldus, Germany). We extracted the time spent exploring the two objects, defined as biting or sniffing at distance of <2 cm, and calculated the recognition index, defined as time spent exploring the novel object relative to the total exploration time.

## 1.8 Full body perfusion and brain sectioning

Twenty-eight days after sham or TBI procedures, we euthanized all rats by transcardiac perfusion ^1, 2^. Briefly, we exsanguinated the animals using ice-cold phosphate-buffered saline (PBS) followed by perfusion of freshly prepared ice-cold 4% paraformaldehyde (PFA) (Sigma-Aldrich, Germany). Immediately after fixation, we carefully harvested the brains, postfixed them for 12 hours in 4% PFA + 15% sucrose and further dehydrated them for 48–72 hours in 30% sucrose. We then quick-froze the brains in dry ice and stored them at -80 °C until further use. Later, full-brain serial sectioning was performed in stereological fashion at 40 μm thickness using a freezing‑stage-equipped microtome (Leica).

## 1.9 Diffuse axonal injury determination

*Amyloid precursor protein staining of axonal bulbs:* We selected the corpus callosum for its especially high vulnerability to shearing, straining, and compression TBI forces, similar to that of the cerebral cortex. In fact, in parasagittal white matter of the cerebral cortex and corpus callosum, large numbers of damaged axonal projections are found after TBI due to deformation and disruption of the neurofilament subunits within the intra-axonal and intracellular cytoskeleton ^8, 97, 8^. To evaluate the effect of CLAS on DAI in white matter tracts, we performed unbiased immunohistochemistry and stereology to quantify the numbers of APP-stained axonal swellings or bulbs on the anterior portion of the corpus callosum in 40 μm coronal brain sections. Briefly, all sections were immersed in 10 mM citrate buffer and placed in water bath at 80°C for 30 min for antigen retrieval and then allowed to cool to room temperature for 10 min. Next, sections were washed in PBS, and endogenous peroxidase activity was quenched with 0.6% hydrogen peroxide in PBS for 30 min. After blocking of nonspecific binding sites with 5% normal goat serum (Sigma-Aldrich, Germany) in tris-buffered saline (TBS) containing 0.25% Triton-X (TBST) for 60 min, the slides were incubated with a primary beta-amyloid polyclonal antibody (cat#: 512700 (CT695), Invitrogen, USA) in a 1:1000 dilution in TBST at 4°C for 48 hours. After TBS/TBST washings, the sections were incubated with goat anti-rabbit secondary antibody (biotinylated, BA‑1000, VectorLaboratories, USA) in a 1:500 dilution in TBST with 2% normal goat serum at room temperature for 4 hours. We then incubated in ABC-Elite kit (PK6105, VectorLaboratories, USA) in a 1:100 dilution in TBST on ice for 1 h. Lastly, we washed all sections in tris-buffer (TB) for 10 min and developed the signal in 0.025% 3,3′‑diaminobenzidine + 0.01% H2O2 in TB with shaking for 8–10 minutes. After completion, sections were washed in PBS, mounted on glass slides, and dried overnight. Once dry, the slides were dipped for 3 min each in 70%, 95%, 100%, and 100% ethanol solutions, followed by Roticlear (Carl Roth, Germany). Finally, the slides were cover-slipped with Rotimount (Carl Roth, Germany) for microscopy and stereological quantification.

*Stereological estimates of APP immunoreactive axonal bulbs*: All stereological estimations were performed by an experimenter blinded to the experimental group. The optical fractionator technique was used to count a systematic random sample of positively stained axonal bulbs within the anterior part of the corpus callosum in collections from two defined adjacent brain regions containing 6 sections each. In brief, we outlined the corpus callosum from each coronal section at low power (4×) using an Axio Imager M2 microscope (Karl Zeiss, Jena, Germany), fitted with a mechanical specimen stage (75x50 mot, CAN (D) for Axio Imager). Next, we systematically counted bulbs at high magnification (63×, oil immersion), of sites within the region examined (Stereo Investigator 10.5 software, MBF Bioscience, USA). To quantify APP, a 200 × 200 μm sampling grid and a 100 × 100 μm counting frame were used. Nonaxonal DAB-stained structures (e.g., DAB-positive red blood cells) were not selected. These parameters allowed reasonable accuracies in cell counts, as determined by Gundersen’s coefficients of error between 5% and 15%.

## 1.10 Myelin integrity determination

*Myelin basic protein staining:* We assessed myelin integrity of axonal projections in the corpus callosum with specific immunohistochemistry against myelin basic protein (MBP), which constitutes one of the most abundant proteins in the myelin sheath, along protocols previously described. We used a similar DAB immunohistochemistry protocol as the one described above for APP detection, except for the antigen retrieval step. Briefly, non-TBI and TBI brains were cryosectioned into 40 µm coronal sections of the rostral portion of the corpus callosum and then DAB-immunostained against MBP using a mouse monoclonal anti-MBP primary antibody ((F-6) sc-271524, Santa Cruz Biotechnology, USA) in a 1:500 dilution in TBST followed by a biotinylated goat anti-mouse secondary antibody (A16076, Invitrogen, USA) in a 1:1000 dilution in TBST. The stained tissue was cover-slipped for microscopy and optical density quantification.

*Optical density quantification of MBP staining:* We used the bright field microscope Axio Imager M2 (Karl Zeiss, Jena, Germany) to acquire images from the corpus callosum in both hemispheres. The region of interest was the corpus callosum in the hemisphere with the highest signal intensity for quantification. We obtained full-section images with a 2.5 × objective. All images were processed in ImageJ (Fiji) using the Hematoxilin-Eosin Diaminobencidine (H&E DAB) vector. We then measured the average colour intensity, which is proportional to the concentration of the staining in the corpus callosum. To correct for background noise, we subtracted the intensity in the striatum. Following the Lambert-Beer law, we calculated the optical density with the formula

OD = log(255/mean_intensity_CC) – log(255/mean_intensity_striatum).

## 1.11 Detailed statistical procedures

Two-way repeated measures ANOVA (RM‑ANOVA, adjusted using the Greenhouse-Geisser correction when necessary) or mixed models were used to assess treatment effects on NREMS duration or fragmentation and SWA time courses. Significant hypothesis-driven main effects or interactions were subjected to post hoc assessments using Dunnett’s multiple comparisons tests. We analysed the behavioural performance by comparing each group’s average recognition index to chance level, a recognition index of 0.5, with a one-sample t-test. A significance level of p <.05 was used for all statistical analyses. For the analysis of histopathological measures, we used Bayesian approaches. The number of APP+ axonal bulbs was analysed using a Bayesian Negative Binomial Regression (brms package for R, version 2.19). The prior for this model was established with the coefficients resulting from a Poisson model as the expected values and a student distribution with the overall data’s standard deviation set as the dispersion value; this provided wide distributions and therefore a more naïve prior. To analyse MBP optical density values, we set up a Bayesian linear regression using a prior with coefficients from the expected values, obtained from a linear model, following a student distribution set as before. Spearman’s correlations were tested between SWA and number of APP+ axonal bulbs, and between SWA and MBP optical density values, as these relationships may not necessarily move in the same direction at a constant rate.

1. **Supplementary figure**


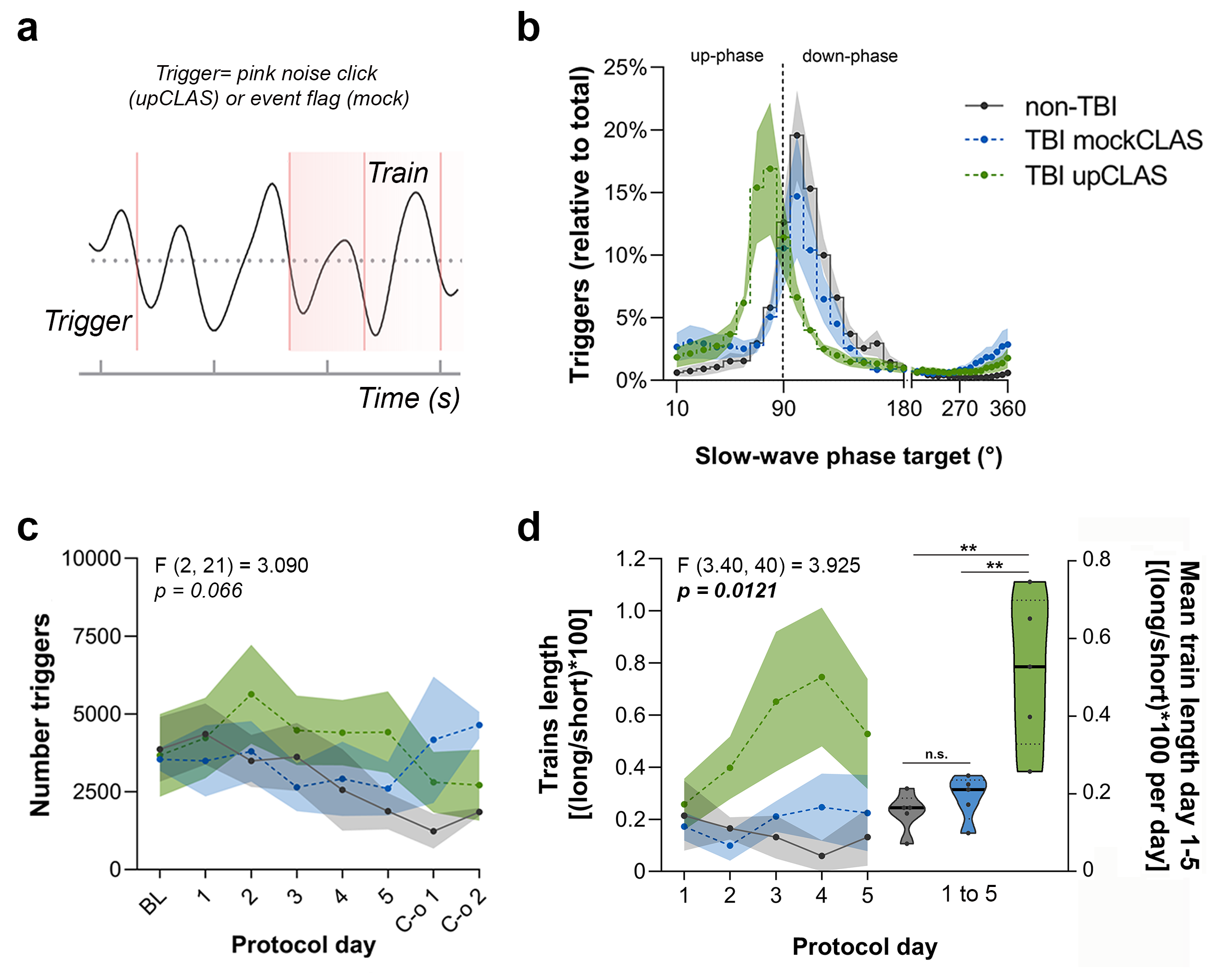


**Figure S1. Characterization of triggers’ phase-targeting accuracy, number and trains across groups. a)** Schematic representation of one trigger or flag (solo vertical red line), and of a sequence of triggers – train – (pink shaded area) in a period of time. **b)** Percentage of triggers distribution across up- and down- phase relative to total targets for each group validates the majority of TBI upCLAS triggers targeted the up-phase of the ongoing slow waves. **c)** Average number of total daily triggers (or flags) per group was not significantly different across stimulation days (One way ANOVA: F (2, 21) = 3.090, *p* = 0.0666). **d)** Up-phase targeted CLAS in TBI progressively increases the ratio of long trains (trains containing 6-10 triggers) over short ones (trains containing 1-5 triggers) during stimulation days, with a distinct peak at day 4 (Two way ANOVA: F (3.397, 40.28) = 3.925, days’ effect *p* = 0.0121), resulting in an overall (days 1-5) significantly increased ratio in the TBI upCLAS group compared to controls (One-way ANOVA: F(2,12) = 13.61, *p*=0.0008; Tukey’s multiple comparisons test: non-TBI v. TBI upCLAS: ***p* = 0.0013; TBI mockCLAS v. TBI upCLAS: ***p =* 0.0030). Mock interventions do not elicit changes between healthy (non-TBI) and TBI rats (TBI mockCLAS) (non-TBI v. TBI mockCLAS: n.s., *p* = 0.8751). s= seconds, BL= baseline, C-o 1-2: carry over day 1-2.

1. **Supplementary references**

1. Büchele F, Morawska MM, Schreglmann SR, et al. Novel Rat Model of Weight Drop-Induced Closed Diffuse Traumatic Brain Injury Compatible with Electrophysiological Recordings of Vigilance States. J Neurotrauma. 2016 Jul 1;33(13):1171-80.

2. Morawska MM, Buchele F, Moreira CG, Imbach LL, Noain D, Baumann CR. Sleep Modulation Alleviates Axonal Damage and Cognitive Decline after Rodent Traumatic Brain Injury. J Neurosci. 2016 Mar 23;36(12):3422-9.

3. Moreira CG, Hofmann P, Mullner A, et al. Down-phase auditory stimulation is not able to counteract pharmacologically or physiologically increased sleep depth in traumatic brain injury rats. J Sleep Res. 2022 Dec;31(6):e13615.

4. Miladinović Đ, Muheim C, Bauer S, et al. SPINDLE: End-to-end learning from EEG/EMG to extrapolate animal sleep scoring across experimental settings, labs and species. PLOS Computational Biology. 2019;15(4):e1006968.

5. Mölle M, Eschenko O, Gais S, Sara SJ, Born J. The influence of learning on sleep slow oscillations and associated spindles and ripples in humans and rats. Eur J Neurosci. 2009 Mar;29(5):1071-81.

6. Blaser R, Heyser C. Spontaneous object recognition: a promising approach to the comparative study of memory. Front Behav Neurosci. 2015;9:183.

7. Ennaceur A, Delacour J. A new one-trial test for neurobiological studies of memory in rats. 1: Behavioral data. Behav Brain Res. 1988 Nov 1;31(1):47-59.

8. Meythaler JM, Peduzzi JD, Eleftheriou E, Novack TA. Current concepts: diffuse axonal injury-associated traumatic brain injury. Arch Phys Med Rehabil. 2001 Oct;82(10):1461-71.

9. McAllister TW. Neurobiological consequences of traumatic brain injury. Dialogues Clin Neurosci. 2011;13(3):287-300.
